# Pyriproxyfen Contamination in *Daphnia magna*: Identifying Early Warning Biomarkers

**DOI:** 10.3390/jox14010013

**Published:** 2024-02-02

**Authors:** Beatriz Salesa, Javier Torres-Gavilá, María Dolores Ferrando-Rodrigo, Encarnación Sancho

**Affiliations:** 1 Biomaterials and Bioengineering Lab, Centro de Investigación Traslacional San Alberto Magno, Universidad Católica de Valencia San Vicente Mártir, 46001 Valencia, Spain; 2 Instituto de Investigación en Medio Ambiente y Ciencia Marina (IMEDMAR-UCV), c/Guillem de Castro 94, 46001 Valencia, Spain; javier.torres@ucv.es; 3 Laboratory of Ecotoxicology, Functional Biology and Physical Anthropology Department, Faculty of Biology, University of Valencia, Dr. Moliner 50, 46100 Valencia, Spain; dolores.ferrando@uv.es (M.D.F.-R.); encarna.sancho@uv.es (E.S.)

**Keywords:** pyriproxyfen, *Daphnia magna*, early biomarkers, gene expression, biochemicals

## Abstract

Pyriproxyfen is an insecticide currently employed in numerous countries for the management of agricultural and indoor pests. Several studies indicate that this insecticide has been detected in multiple rivers, with concentrations reaching as high as 99.59 ng/L in the Júcar River in Spain. Therefore, the determination of some biochemical and genetic effects of this insecticide on aquatic organisms could serve as an early warning mechanism to identify potential disruptions in various biomarkers. Based on this, *Daphnia magna* organisms were exposed to pyriproxyfen sublethal concentrations for 21 days. Some biochemical parameters, including cholesterol, triglycerides, glucose, lactate, and LDH activity, were determined. Additionally, some genetic biomarkers associated with oxidative stress, heat shock proteins, lipid metabolism, hemoglobin, metallothioneins, and vitellogenin synthesis were evaluated in daphnids exposed to the insecticide for 21 days. LDH activity increased significantly in those daphnids exposed to the highest insecticide concentration (14.02 µg/L), while cholesterol levels decreased significantly. In contrast, glucose, total proteins, and triglycerides remained unaffected in *D. magna* exposed to pyriproxyfen. On the other hand, exposure to the insecticide led to notable alterations in gene expression among individuals. Specifically, genes associated with lipid metabolism and reproduction exhibited a significant reduction in gene expression. F*abd* expression was decreased by approximately 20% in exposed daphnids, while *vtg* expression was suppressed as much as 80% when compared to control values. Furthermore, it was observed that the *hgb1* and *hgb2* genes, associated with hemoglobin synthesis, exhibited significant overexpression. Notably, the dysfunction observed in both hemoglobin genes was linked to an increase in pigmentation in *Daphnia magna* during the course of the experiment. These alterations in gene expression could serve as effective indicators of early contamination even at low pesticide concentrations.

## 1. Introduction

Pyriproxyfen, chemically known as 2-[1-methyl-2-(4-phenoxyphenoxy) ethoxy] pyridine, is a powerful insecticide functioning as an insect growth regulator, and it has a broad range of applications for pest control. It is extensively employed in various public health programs, serving as an alternative to organophosphate and pyrethroid pesticides [1]. In fact, it is one of the most efficacious juvenile hormone analogs [2] due to its development and larvae maturation inhibition effect [3], use as a juvenile hormone III mimic [4], in preventing insect hatching success [5], disrupting larval development [6], or inducing morphological abnormalities in adult insects, which decreases their reproductive capacity [7], among others. The effectiveness of its active ingredient was assessed to determine its safety for use as an insecticide in drinking water [8], ensuring minimal adverse effects on non-target aquatic insects and the surrounding environment [9,10,11,12].

Pyriproxyfen, known for its relative environmental stability and lipophilic properties (octanol/water partitioning coefficient of Kow 5.6), may have a significant impact in freshwater environments which are close to agricultural areas [13]. Concentrations of this insecticide between 37.74 ng/L and 99.59 ng/L had been detected in western Mediterranean European rivers, such as the Ebro [14] or Júcar [15], derived from agriculture uses. 

According to the World Health Organization (WHO) [8], safe concentration levels for this insecticide are set at 10 µg/L for drinking water sources and 100 mg/L for bodies of water. Furthermore, various studies have shown that the effective concentrations utilized for controlling mosquito pests can result in detrimental impacts on a range of terrestrial invertebrates like honeybees, as well as aquatic invertebrates such as amphipods, crabs, and daphnids, affecting both embryos and adult individuals [16,17,18,19,20].

*Daphnia magna* hold a crucial role as a keystone species in aquatic ecosystems, serving as primary consumers of algae and providing a vital food source for larger invertebrates and fish [21]. Disruptions in the population of primary consumers can contribute to environmental issues (i.e., algal blooms, reduced fish populations, and overall disruption of the aquatic ecosystem).

Invertebrates’ early exposure to an endocrine disruptor may result in long-term effects in their endocrine systems, potentially leading to reproductive effects in adult individuals [22,23]. Reproduction in parthenogenetically populations such as *D. magna* could be seriously affected by pyriproxyfen exposure, resulting in an elevated male production [24]. Olmstead and LeBlanc [25] determined pyriproxyfen’s male production EC_50_ in *D. magna*, about 170–310 pM (55–100 ng/L). Elevated male production in Daphnia species leads to a decrease in the production of parthenogenic females, a decrease in the population growth rate and, ultimately, in the overall population abundance [25]. 

Taking into account pyriproxyfen residues resulting from agricultural applications in water during the initial weeks post-application, these levels could potentially reach concentrations that negatively impact invertebrate populations and other primary consumers residing in rice fields, floodwaters, freshwater ponds, and similar environments. Biochemical parameters encompass a wide range of responses, and they can be highly sensitive to various pollutants. The selection of key general metabolites associated with Daphnia’s energy metabolism, such as proteins, lipids, glucose, and the lactate pathways, is a straightforward approach, yielding a rapid response for assessment [26]. These biomolecules are crucial for the proper development of an individual, and deviations in their concentrations could be attributed to stress induced by the presence of a contaminating agent [27]. Moreover, an examination of genes associated with various processes, including xenobiotic detoxification pathways (such as metallothionein synthesis), responses to stressors (heat shock protein synthesis), oxidative stress management (involving glutathione-S-transferase and catalase), reproduction (vitellogenin), the regulation of fatty acids (acyltransporter protein synthesis), and hemoglobin production, could serve as an early warning system for assessing the health status of individuals and, ultimately, entire populations.

Rider et al. [28] established that the accumulation of hemoglobin in daphnids is a significant adaptive response that is governed by at least two separate molecular pathways: one driven by endocrine influences activated by terpenoid hormones and another associated with oxygen sensing, which involves the hypoxia-inducible factor. Research into environmental signals capable of triggering neuro-endocrine cascades that govern various physiological processes has revealed that the accumulation of hemoglobin and the production of male offspring are concurrently influenced by the crustacean terpenoid hormone methyl farnesoate, as well as several synthetic analogues like pyriproxyfen. While sublethal effects do not lead to immediate mortality, they have adverse effects on the individual. The physiological changes observed in response to environmental pesticide levels, particularly in invertebrates other than insects, not only offer insights into the ecotoxicological implications of pollutants at biological levels but can also serve as a model for understanding the aquatic toxicity of vertebrates. 

The primary goal of this study was to investigate the physiological responses of *Daphnia magna* individuals when exposed to chronic exposure to the insecticide pyriproxyfen, a potent endocrine disruptor in insects. This research was conducted under controlled laboratory settings. Biochemical parameters associated with intermediate metabolism were utilized as critical indicators of insecticide toxicity at the individual level. Additionally, the study explored the expression of certain genes linked to oxidative stress responses (*gst* and *cat* genes), lipid metabolism (*fabd* gene), hemoglobin (*hb1* and *hb2* genes), vitellogenin (*vtg 1* and *vtg 2* genes), heat-shock protein genes (*hsp70* and *hsp 90*), and metallothioneins (*mt-a*, *mt-b*, *mt-c* genes), which were considered as potential tools for the early detection of individual dysfunction. The parameters selected could prove to be valuable as a diagnostic test for assessing the well-being of aquatic invertebrates following pesticide spray operations.

## 2. Materials and Methods

### 2.1. Daphnia magna Culture

*Daphnia magna* clon K6 [29] were maintained in our laboratory following OECD [30] recommendations for more than 20 years. The culture was set in a controlled climate chamber with a room temperature of 22 ± 1 °C, and a photoperiod of 16:8 h light/dark. Daphnids were kept in well-oxygenated tap water with a pH of 7.9 ± 0.2, alkalinity of 4.1 mmol/L, total hardness of 181.8 ± 18.8 mg/L as CaCO_3_, and oxygen saturation of more than 90%. The population density was maintained below 40 animals per liter. *Nannochloropsis oculata* algae at a concentration of 5 × 10^5^ cells/mL was used as food for daphnids and were cultivated in our laboratory using Bold’s Basal Medium [31] under constant illumination [32,33]. The daphnids used in the experiments were only females, as they reproduced by parthenogenesis in good environmental conditions [33,34].

### 2.2. Test Chemical and Solutions

Technical product pyriproxyfen (CAS number 95737-68-1, >97% purity), chemical name: 4-phenoxyphenyl (RS)-2-(2-pyridyloxy) propyl ether was provided by The Plant Protection Company S.L (Proplan, Madrid, Spain). 

Pyriproxyfen experiments were conducted in our laboratory using five different test concentrations: 3.14, 4.74, 6.12, 8.40, and 14.02 µg/L. These concentrations corresponded to 1/107, 1/71, 1/55, 1/40, and 1/24 of the EC_50_—48 h value [35]. Ethanol was used as the pyriproxyfen solvent with a maximum concentration of 0.28 µL/L. Previous experiments have confirmed that this ethanol concentration is safe for *Daphnia magna*, and no adverse effects were observed in this species [36]. The pyriproxyfen stock solutions were freshly prepared daily using a renewal medium.

### 2.3. Experimental Design

In the experimental setup, an individual adult female was randomly selected from the general culture. All her offspring from a single brood were then placed in a separate culture, where they were raised under the same environmental conditions as the general culture. Starting from the third brood onward, the neonates were utilized in the experiments.

For each experiment, 40 neonates (≤24 h old) were randomly distributed into individual 3 L glass beakers. The experimental medium and food (*N. oculata* at a concentration of 5 × 10^5^ cells/mL) were consistently maintained by transferring the *Daphnia* to fresh test solutions or control water every day. The experimental water and food were the same as those used in the general *Daphnia* culture, as previously described, and the experimental maintenance conditions in the climate chamber were in accordance with the conditions mentioned in Section 2.1 (*Daphnia magna* culture). These experiments were conducted for 21 days using five different concentrations of pyriproxyfen, in addition to a control group, all under these specified conditions.

Upon reaching reproductive maturity, the offspring were regularly extracted from the containers during the daily medium renewal to ensure that only adult individuals remained in each culture. After 21 days of exposure to these conditions, each adult *Daphnia* was carefully transferred to a labeled 1.5 mL Eppendorf tube. In order to guarantee complete dryness before preservation, any residual medium was removed. Subsequently, the labeled tubes containing the Daphnia specimens were promptly frozen using liquid nitrogen and stored at a temperature of −80 °C until further analysis.

### 2.4. Biochemical Analysis

For each pesticide concentration plus control group, 6 replicates (2 individuals/replicate) were performed. Test organisms were placed in microcentrifuge tubes with 30 µL of phosphate buffer (pH 7.2) and homogenized on ice. Subsequently, the homogenate was centrifuged (6 min, 1400× *g*) and the supernatant transferred to a clean tube preserved on ice for its immediate use. All reactions took place at laboratory temperature (25 °C).

Bradford Protein Assay reagent (Bio-Rad, Hercules, CA, USA) according to Bradford (1976) was used to determine the protein content in the samples using bovine serum albumin (Sigma-Aldrich, Zwijndrecht, Switzerland) as standard. Total cholesterol, triglycerides, glucose, and lactate dehydrogenase (LDH) were determined by using commercial Spinreact’s kits (refs. 001090, 1001310, 1001200, and 1001260, respectively) following the manufacturer’s protocol. 

All the assays were adjusted for measurement in microplates. The contents of each sample were determined spectrophotometrically using a Varioskan microplate reader (ThermoScientific, Waltham, MA, USA), at their respective absorbance wavelengths. The data obtained were subsequently normalized to protein content and expressed for each individual *Daphnia*.

### 2.5. RNA Isolation and Quantitative RT-PCR

To analyze the gene expression, a total of 6 replicates were performed. In each replicate (1 individual per replicate), manual homogenization was carried out using the buffer provided by the RNA purification kit (Norgen, Thorold, ON, Canada). The extraction process was conducted in accordance with the manufacturer’s protocol. The concentrations and quality of the extracted RNA were assessed using a Nanodrop™ One instrument (ThermoScientific™, Waltham, MA, USA). Finally, 1 μg of RNA was used by means of the PrimeScript™ RT (Perfect Real Time) kit, reverse transcribing for the cDNA synthesis (Takara Bio Inc., Otsu, Japan).

To assess the expression of the genes selected, RT-PCR was conducted using TB Green Premix Ex Taq (Takara Bio Inc., Otsu, Japan) as the reagent in the 384 QuantStudio 5 real-time PCR detection system (ThermoScientific™, Dreieich, Germany). The thermocycling profile consisted of an initial denaturation step at 95 °C for 10 min, followed by 40 amplification cycles at 95 °C for 15 s, 60 °C for 30 s, and 72 °C for 15 s. GAPDH (glyceraldehyde-3-phospate dehydrogenase) was used as the reference gene. A melting curve analysis was carried out to confirm the unique amplification of the selected genes.

The primer sequences employed in the study are presented in Table 1. We used QuantStudio™ Design and Analysis Software 1.5.1 (ThermoFisher, Waltham, MA, USA) to analyze the data obtained from the real-time PCR analysis.

### 2.6. Statistical Analysis 

GraphPad Prism 7 software (GraphPad Software, Solana Beach, CA, USA) was used for statistical analyses. Kolmogorov–Smirnov and the Levene tests were used in a priory test. Significant differences between groups were assessed using one-way ANOVA, followed by Dunnett’s multiple tests as a post hoc analysis. The significance level was predetermined at *p*-values < 0.05.

## 3. Results and Discussion

After 21 days of *Daphnia magna* chronic pyriproxyfen exposure, no mortality was observed, and the survival rate remained at 100% in both the control groups and the exposed daphnids.

**Figure 1:**
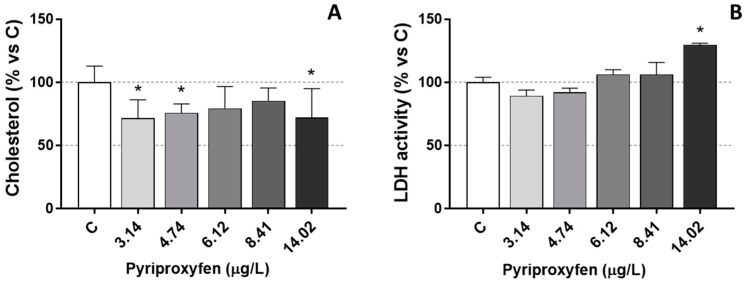
LDH activity (**A**) and cholesterol levels (**B**) in *D. magna* exposed to pyriproxyfen for 21 days, compared (%) to control group C. Results are mean ± SEM (* *p* < 0.05).

Lactate dehydrogenase activity (LDH), as well as cholesterol levels in pyriproxyfen-exposed *D. magna*, are shown in Figure 1. As we can see in Figure 1A, LDH activity increased significantly (*p* < 0.05) in those daphnids exposed to the highest pesticide concentration (14.02 µg/L), while cholesterol levels decreased significantly (*p* < 0.05) (Figure 1B).

**Figure 2:**
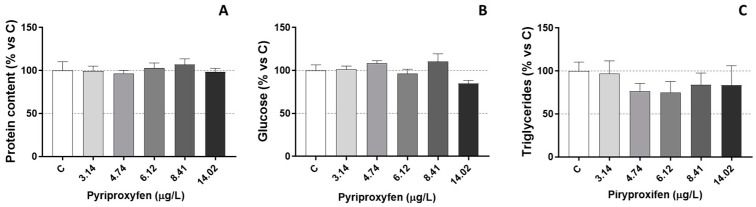
Protein content (**A**), glucose levels (**B**), and triglyceride levels (**C**) in *D. magna* exposed to pyriproxyfen for 21 days, compared (%) to control group C. Results are mean ± SEM.

In contrast, glucose, total proteins, and triglycerides (Figure 2) remained unaffected in those daphnids exposed to pyriproxyfen. Therefore, the results suggest that the energy reserves of *D. magna* were not significantly affected by the selected pyriproxyfen concentrations during the 21-day period, and the findings do not indicate a substantial toxic stress response.

The increase in LDH activity observed at the highest exposure concentration may be linked to increased resource demand. Wu and Lam [40] previously reported a negative correlation between LDH activity and ambient oxygen levels in *D. magna*, suggesting a potential biochemical adjustment in response to hypoxic conditions. LDH is a cytoplasmic enzyme found in numerous tissues, and its elevation, following intoxication, could signify both damage and inadequate oxygen intake, as well as an additional energy requirement to meet the heightened energy demands of stressed animals. In fact, a transient increase in LDH activity could indicate the induction of anaerobic glycolysis to fulfill the heightened energy demands. In vertebrates like fish, increased anaerobic metabolism is a rapid and clear response to the energy depletion caused by oxygen deprivation [41]. A reduction in lipid reserves is typically associated with an increase in energy demand in pesticide-exposed animals, and lipid catabolism might be an outcome of a toxicant-induced stress situation. However, in the present study we did not find a mobilization of lipid reserves (as triglycerides) and only cholesterol levels decreased in our exposed daphnids. Decreases in both cholesterol and triglycerides were observed in *D. magna* because of chronic exposure to other pesticides, such as the fungicide tebuconazole [42].

**Figure 3:**
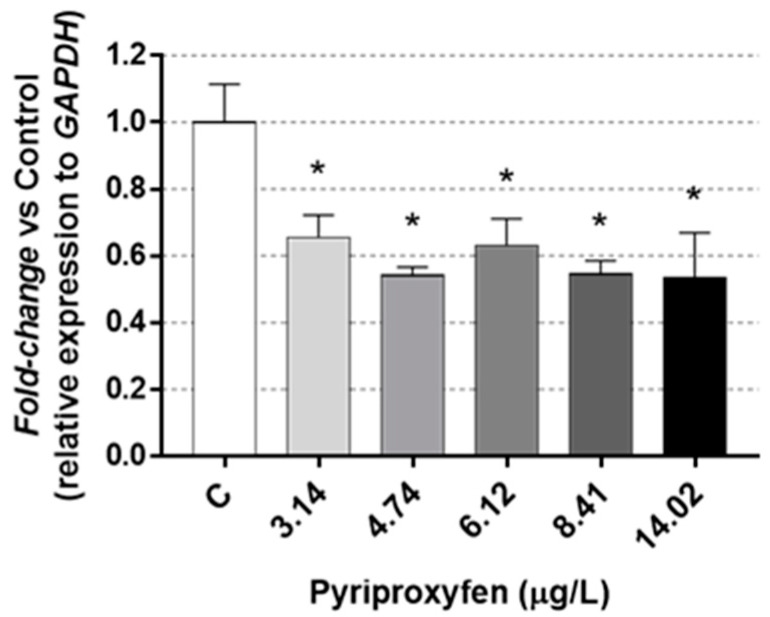
Results in *fabd* gene expression in *D. magna* exposed to pyriproxyfen for 21 days, compared (%) to control group C. Results are mean ± SEM (* *p* < 0.05).

The *fabd* gene, linked to lipid metabolism (Figure 3), showed a significant under-expression, with a reduction exceeding 40% compared to the control, in those daphnids which were exposed to all the selected pyriproxyfen concentrations. So, a drop in this gene expression would lead to an alteration in the storage of lipid reserves, which were not found in the present study.

Very little is known about the mechanisms by which toxicants can disrupt lipid accumulation in invertebrates. Knowledge about invertebrate lipid metabolism and their regulation needs further studies, as suggested by Fuertes et al. [43], in *D. magna*.

Some other studies have reported the overexpression of this gene in *Daphnia magna* because of exposure to substances like triphenyl phosphate [44]. In contrast, Seyoum et al. [38] investigated lipid metabolism and related genes in *D. magna* under phthalate exposure and found that the expression of *fabd* remained unchanged. These varying responses underscore the complexity of how different chemicals can influence gene expression in *D. magna*.

**Figure 4:**
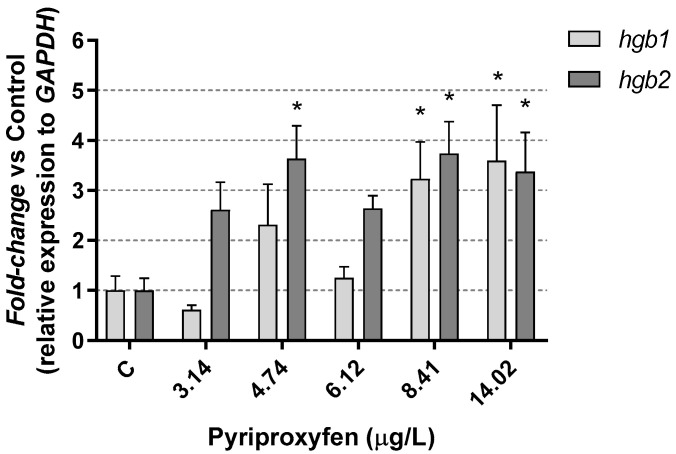
Hemoglobin *hgb1* and *hgb2* gene expression results in *D. magna* exposed to pyriproxyfen for 21 days, compared (%) to control group C. Results are mean ± SEM (* *p* < 0.05).

Moreover, it is noteworthy that pesticides can influence the structural integrity of gills, potentially reducing their efficiency as respiratory organs. In the current study, an increase in *Daphnia magna* hemoglobin (*hgb1* and *hgb2)* gene expression was observed after exposure to the highest pyriproxyfen concentrations (Figure 4). In fact, at the highest concentration tested, a significant (*p* < 0.05) overexpression, exceeding a five-fold change, was identified. This insecticide induced the overexpression of *hgb1* and *hgb2* gene products. Such inductions could function as a defense mechanism, as described by Eytcheson and LeBlanc [45]. The authors demonstrated that exposing *Daphnia magna* to a low concentration of pyriproxyfen (96 ng/L) for 48 h triggered the activation of hemoglobin synthesis. This adaptive response assisted in reducing the toxicity of sodium nitrite present in the environment, acting as a protective measure against this pollutant.

It is well-established that stress represents a mechanism of physiological compensation that an organism adopts in response to a challenging physical or chemical factor, in our case, the insecticide pyriproxyfen. Our findings are like those found by Rider et al. [28], who exposed *Daphnia magna* and *Daphnia pulex* individuals to methyl farnesoate as well as to pyriproxyfen (at concentrations ranging from 23 to 64 ng/L). They observed the stimulation of neuroendocrine cascades, leading to an increase in hemoglobin levels. This physiological response was accompanied by a visible change in the color of the individuals, transitioning from a pale hue to shades of yellowish and reddish.

**Figure 5:**
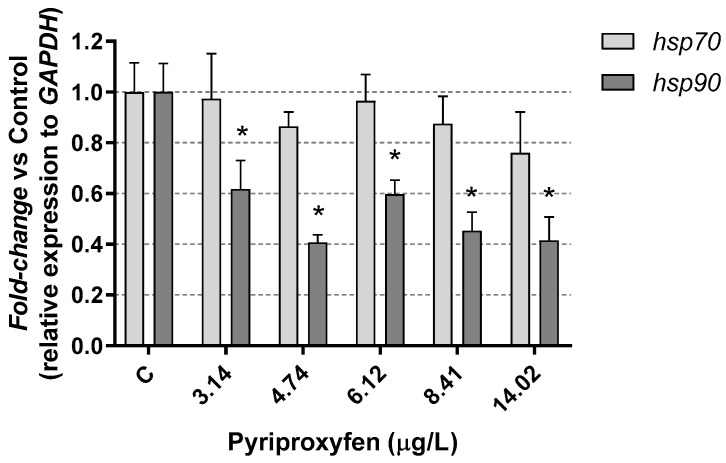
Heat-shock protein *hsp70* and *hsp90* gene expression results in *D. magna* exposed to pyriproxyfen for 21 days, compared (%) to control group C. Results are mean ± SEM (* *p* < 0.05).

In response to exposure to the insecticide, a reduction in heat-shock protein (*hsp90)* gene expression, approximately 40%, was observed in the exposed *Daphnia*, with no changes found in *hsp70* gene expression (Figure 5). Similar results were found by Silva et al. [46] in *Daphnia magna* exposed to the pesticide carbendazim. The authors suggested that the repression of heat-shock proteins might indicate that the mechanisms for cellular repairing damage could be compromised.

However, it is important to note that other studies have reported an overexpression of the *hsp90* gene in *Daphnia magna* when exposed to the flame-retardant triphenyl phosphate for 21 days [44]. Exposure to microplastics also appears to induce the overexpression of these genes, as indicated by Liu et al. [47], in *Daphnia pulex* over the exposure period.

The expression of *hsp* genes can vary not only due to the presence of xenobiotics but also due to various biological parameters, such as the presence of predators [47] or physical parameters like temperature [48]. In this context, our results are similar to those found by Kim et al. [49], who observed changes in *hsp90* expression induced by exposure to mercury. As explained by the authors, the expression patterns of these genes can fluctuate based on exposure time and other factors.

**Figure 6:**
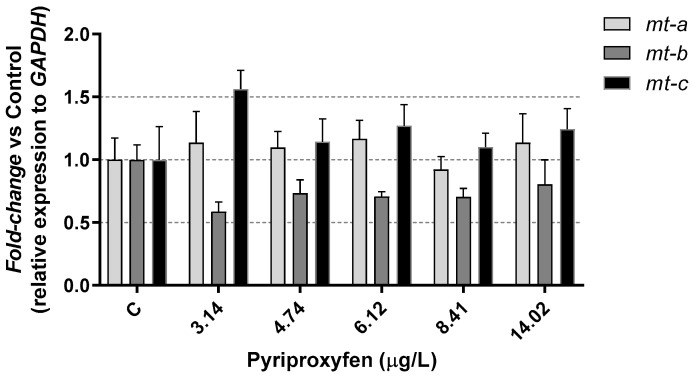
Metallothioneins results in *D. magna* exposed to pyriproxyfen for 21 days, compared (%) to control group C. Results are mean ± SEM.

In our study, no significant (*p* > 0.05) alterations were observed in *D. magna* metallothioneins *mt-a*, *mt-b*, and *mt-c* gene expression following exposure to pyriproxyfen (Figure 6). Many studies have established a connection between the suppression of these genes with increased toxicity resulting from various types of toxic factors, including metals or pesticides. This inhibition often leads to oxidative stress and is associated with metabolic imbalances [50,51,52].

Furthermore, products that are generally characterized as non-endocrine active compounds, such as perfluorinated alkyl substances, have been shown to inhibit the expression of these three genes in *Daphnia magna* [53]. These results highlight the diverse responses of Daphnia to different chemical compounds and their potential effects on gene expression.

In relation to oxidative stress, gluthathione-s-transferase (*gst)* gene and catalase (*cat)* gene were studied in *D. magna* after 21 days of exposure to pyriproxyfen. No significant (*p* > 0.05) alterations were observed in the expression of the *gst* gene, whereas the expression of the *cat* gene was significantly (*p* < 0.05) reduced (Figure 6). These genes are involved in antioxidant pathways and encode key antioxidant enzymes, including glutathione-S-transferase (gst) and catalase (cat), which are utilized by many aquatic organisms to mitigate the harmful effects of reactive oxygen species (ROS) [54].

It is important to note that different xenobiotics can produce varying effects on these genes in *Daphnia*. For instance, some studies have shown that phthalates inhibited the expression of both *gst* and *cat* genes in *Daphnia magna* [38,55], while the fungicide prochloraz did not significantly reduce the gene expression of *gst* and *cat* in the same species [50]. On the other hand, a decrease in *gst* and an increase in *cat* genes expression were found in *D. magna* exposed to the fungicide boscalid [56]. Kim et al. [49] observed an increase in GST activity in *Daphnia pulex* at a concentration of 2 µg/L pyriproxyfen. In our study, we did not examine GST activity, and the unaltered expression observed could suggest that the antioxidant response remained unaffected or disrupted. Further studies would be needed to provide a more comprehensive understanding of this response.

**Figure 7:**
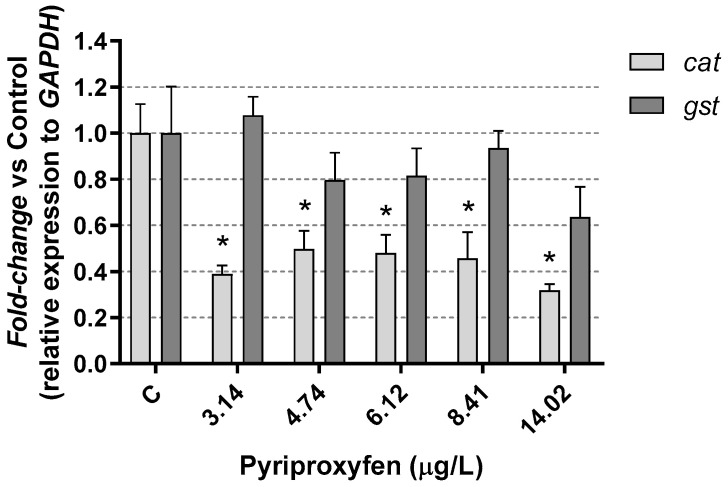
Oxidative stress (*cat* and *gst*) results in *D. magna* exposed to pyriproxyfen for 21 days, compared (%) to control group C. Results are mean ± SEM (* *p* < 0.05).

Oxidative cellular stress takes place if the concentration of free oxygen radicals (ROS) exceeds the natural neutralizing capacity of the cell [57]. Liu et al. [45] determined that the presence of microplastics in their environment seems to affect the expression of the stress defense *gst* gene in *D. magna*. The authors indicated that this effect was an age function, with both inhibition and over expression. Inhibition would be in relation to juveniles of 7 and 14 days for the gene *gst*, while overexpression would relate to early individuals (24–96 h) and the oldest individuals (21 days). In the same study, the authors observed that the *cat* gene experienced overexpression, but only after 21 days of exposure. In our results, the expression of *cat* gene was inhibited in daphnids after 21 days of exposure at all the toxicant concentrations tested, as is shown in Figure 7. This suggests a potentially disruptive effect on the response to oxidative stress.

**Figure 8:**
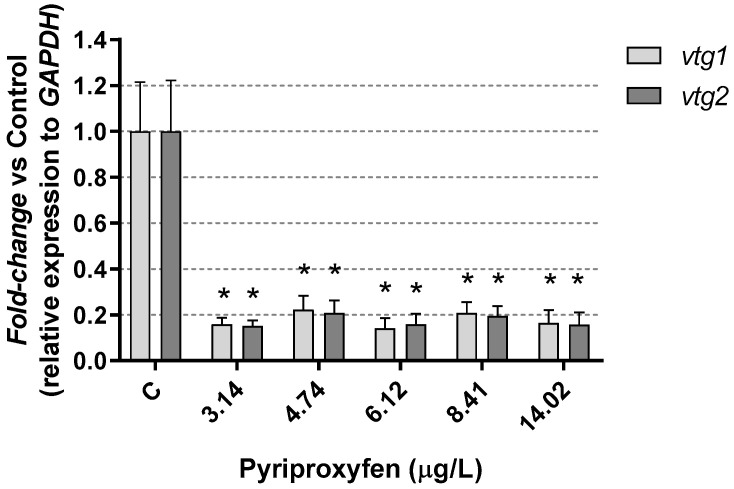
Vitellogenin results in *D. magna* exposed to pyriproxyfen for 21 days, compared (%) to control group C. Results are mean ± SEM (* *p* < 0.05).

Vitellogenin is considered a potential early biomarker which may precede reproductive effects in Daphnia exposed to various xenobiotics. In our study, *vtg1* and *vtg2* gene expressions in *Daphnia magna* were strongly inhibited (>80%) after 21 days of pyriproxyfen exposure (Figure 8). This observation confirms the reproductive impact of pyriproxyfen on the exposed females and underscores its role as an endocrine disruptor.

These results are similar to those found by Seyoum et al. [53] in *D. magna* exposed to alkyl perfluorinated, who associated the reduction in the number of neonates per daphnia joint with the under expression of the *vtg2* gene. Vitellogenin, an essential yolk protein in Daphnia, could be stimulated by anti-ecdysteroid products [58], promoting egg development. However, the results of pyriproxyfen exposure are indicative of a strongly disruptive effect on reproduction. The lowest concentration tested, 3.14 µg/L, caused a depletion of progeny greater than 50% after 21 days, while the time to first brood was significantly increased; additionally, male progeny was observed at all exposure concentrations [35].

## 4. Conclusions

The exposure of *Daphnia magna* to pyriproxyfen altered its metabolism, as indicated by the differential gene expression results. However, all the animals in the study survived the exposure period at all the tested insecticide concentrations. Our findings highlight a clear endocrine-disruptive effect on *D. magna*, not only at the reproductive level, as indicated by the expression of *vtg1* and *vtg2* genes, but also with implications for the maintenance and growth of the individuals. Therefore, we could consider vitellogenin as a potential early biomarker for pyriproxyfen in *Daphnia magna*.

To obtain a better understanding of the minimal effects of pyriproxyfen on aquatic invertebrates, such as daphnids, longer exposure periods and lower concentrations should be considered. These extended assays will be valuable in identifying disruptive effects in the environment and helping to protect invertebrate populations. Invertebrate species, particularly *D. magna*, have been effectively utilized in screening and testing assays for many years. Such applications should now play a crucial role in screening and testing programs for endocrine-disrupting chemicals (EDCs).

## Figures and Tables

**Table 1:** Primer sequences employed for gene-specific real-time RT-PCR.

Gene	Forward	Reverse	Reference
*GAPDH*	TGCTGATGCCCCAATGTTTGTTGT	GCAGTTATGGCGTGGACGGTTGT	[37]
*vtg1*	CCAGCGAATCCTACACCGTCAAG	GAGCCGCACAGACCACAGAG	[38]
*vtg2*	CGTCCGCCACTGGTTGGGTC	GGGGCAGCCAAGACAGAGCG	
*mt-a*	GAGCGCCATGCCAAAATCCC	TCGTCGTTGTAAAATCCGGCT	
*mt-b*	TGGAACCGAATGCAAATGCG	CGGACTTGCATGGACAACTG	
*mt-c*	AAAGTGTGCCCTCGTTGTCA	CTTACAGTCGTCCCCACACG	
*gst*	TCAGGCTGGTGTTGAGTTTG	GAGCAAGCATTTGTGCATCA	
*cat*	TGGCGGAGAAAGCGGTTCAGC	GTGCGTGGTCTCTGGGCGAA	
*hsp70*	CGACGGCGGGAGATACGCAC	CCACGGAAAAGGTCGGCGCA	
*hsp90*	CCCTCTGTGACACTGGTATTGGCA	GCCCATGGGTTCTCCATGGTCAG	
*fabd*	GCCAACTACCTGTATCCTGAATG	GTGGAACGCTCCGCTAACT	
*hb1*	ACAAATTGCTCTGGTTGCCG	AAGGTTTTTGAGTGCCACGT	[39]
*hb2*	TGTTACCACCAGTGTCACCA	TATTCAGGGTGGGCCTTGAT	

## Data Availability

All data generated or analyzed in this study are included in this published article.
